# LXR activation potentiates sorafenib sensitivity in HCC by activating microRNA-378a transcription

**DOI:** 10.7150/thno.45158

**Published:** 2020-07-11

**Authors:** Zhongjie Lin, Shunjie Xia, Yuelong Liang, Lin Ji, Yu Pan, Shi Jiang, Zhe Wan, Liye Tao, Jiang Chen, Chengping Lin, Xiao Liang, Junjie Xu, Xiujun Cai

**Affiliations:** Key Laboratory of Laparoscopic Technology of Zhejiang Province, Department of General Surgery, Sir RunRun Shaw Hospital, Zhejiang University School of Medicine, Hangzhou 310016, China.

**Keywords:** hepatocellular carcinoma, sorafenib resistance, microRNA, IGF1R, XPO5, LXRα

## Abstract

Sorafenib resistance is a major obstacle to the treatment of advanced hepatocellular carcinoma (HCC). MicroRNAs (miRNAs) are multifunctional regulators of gene expression with profound impact for human disease. Therefore, better understanding of the biological mechanisms of abnormally expressed miRNAs is critical to discovering novel, promising therapeutic targets for HCC treatment. This study aimed to investigate the role of miR-378a-3p in the sorafenib resistance of HCC and elucidate the underlying molecular mechanisms.

**Methods**: A novel hub miR-378a-3p was identified based on miRNA microarray and bioinformatics analysis. The abnormal expression of miR-378-3p was validated in different HCC patient cohorts and sorafenib-resistant (SR) HCC cell lines. The functional role of miR-378a-3p and its downstream and upstream regulatory machinery were investigated by gain-of-function and loss-of-function assays *in vitro* and *in vivo*. Interactions among miR-378a-3p, LXRα, and IGF1R were examined by a series of molecular biology experiments. Then, the clinical relevance of miR-378a-3p and its targets were evaluated in HCC samples. HCC patient-derived xenograft (PDX) model was used to assess the therapeutic value of LXRα and its downstream miR-378a-3p.

**Results**: miR-378a-3p expression was frequently reduced in established sorafenib-resistant HCC cell lines. The decreased miR-378a-3p levels correlated with poor overall survival of HCC patients following sorafenib treatment. miR-378a-3p overexpression induced apoptosis in SR HCC cells, whereas miR-378a-3p silencing exerted the opposite effects. IGF1R was identified as a novel target of miR-378a-3p. Furthermore, the primary miR-378 level was not consistent with its precursor miRNA level in SR HCC cells, which was attributed to the downregulation of exportin5 (XPO5) and subsequently reduced nuclear export of precursor miR-378 and restrained maturation of miR-378-3p. In this context, we combined an agonist GW3965 of liver X receptor alpha (LXRα), which functioned as a transcription activator of miRNA-378a, and its activation re-sensitized sorafenib-resistant cells to sorafenib treatment *in vitro* and *in vivo*.

**Conclusions**: Our finding suggested decreased expression of XPO5 prevents maturation of miR-378a-3p, which leaded to the overexpression of IGF-1R and counteracted the effects of sorafenib-induced apoptosis. LXRα was able to activate miRNA-378a-3p transcription in HCC cells and could be a potential combinable treatment strategy with sorafenib to suppress HCC progression.

## Introduction

Despite its rising global incidence and status as the leading cause of cancer death, historically, there has been a dearth of new drug developments for hepatocellular carcinoma (HCC). Sorafenib, an oral multi-kinase inhibitor, has been regarded as the first-line systemic treatment modality in patients with advanced HCC 1. The efficacy of sorafenib results from its activities in targeting cancer cells and cells of the tumor microenvironment: this agent inhibits up to 40 kinases, including angiogenic receptor tyrosine kinases (including VEGF receptors and PDGF receptor-β) and drivers of cell proliferation (such as RAF1, BRAF, and KIT) 2. However, few patients obtain real and long-term benefits from this treatment because of the high rate of resistance to sorafenib therapy^3^. Therefore, clarifying the resistance mechanism is critical to identify new treatment strategies for sorafenib non-responders.

MicroRNAs (miRNAs) are small (around 22 nucleotides), non-coding RNA gene products existing in many organisms and play crucial post-transcriptional regulatory roles in mRNA translation and degradation by binding to the 3′-untranslated region (3′-UTR) of target genes 4. Aberrant expression of miRNAs alters numerous aspects of cellular function such as differentiation, apoptosis, and survival signaling, linking miRNAs to disease pathology 5,6. Several miRNAs have demonstrated to be associated with sorafenib resistance and to function as predictive biomarkers for sorafenib responses in HCC patients. For example, miR-367-3p increases the efficacy of sorafenib to suppress HCC metastasis through altering androgen receptor signals 7. These findings indicate that miRNAs play a critical role in the development of sorafenib resistance.

The biogenesis of miRNA involves multiple steps, including transcription of primary miRNA (pri-miRNA) by RNA polymerase II, cleavage of pri-mRNA to precursor miRNA (pre-miRNA) by Drosha, nucleocytoplasmic export of pre-miRNA by exportin-5 (XPO5), processing of pre-miRNA to mature miRNA by Dicer, and formation of functional RNA-induced Silencing Complex containing Argonaute^8^. Global downregulation of miRNA expression has been observed in many tumors 9. Moreover, systematic evaluation of miRNA levels in cancer cell lines demonstrated that many pre-miRNAs are retained in the nucleus 10, implying that function of the nuclear-cytoplasmic export machinery might be compromised in tumors.

miRNA genes are categorized based on genomic location, including intronic miRNAs in noncoding transcripts, exonic miRNAs in noncoding transcripts, intronic miRNAs in protein‐coding transcripts, and exonic miRNAs in protein‐coding transcripts 11. Both exonic and intronic miRNAs in protein‐coding transcripts share the transcription machinery with their host genes 11, 12. miR-378a (previously known as miR-378) is a small noncoding RNA that regulates gene expression at the posttranscriptional level. The two mature strands, miR-378a-3p and miR-378a-5p, originate from the first intron of the peroxisome proliferator-activated receptor gamma, coactivator 1 beta (ppargc1b) gene encoding PGC-1β 12, 13. Different from other miRNAs, miR-378 possesses its own transcription machinery independent of its host gene 14. A previous study combining database mining, EST extension, and 5′RACE and 3′RACE confirmed that the nuclear receptor Liver X receptor alpha (LXRα) is a transcription factor that regulates miR-378 14.

The insulin-like growth factor (IGF) signaling axis is critical to the growth, development, and maintenance of multiple tissues 15. IGF signaling is particularly important during neonatal and pubertal growth, particularly by simulating cell proliferation and interrupting programmed cell death 15, 16. Recently, IGF signaling was shown to be enriched in tumors with sorafenib-acquired resistance 17, 18, which strongly suggests that IGF signaling may play an important role in conferring drug resistance in human malignancies.

In this study, we aimed to identify miRNAs that regulate sorafenib resistance in HCC by analyzing differentially expressed miRNAs from paired HCC cells and sorafenib-resistant (SR) HCC cells. We found a set of miRNAs, including miR-378a-3p, that were altered in SR cells compared with wild-type (WT) cells. Overexpression of miR-378a-3p by LXRα activation enhanced sorafenib-induced apoptosis in HCC through inhibiting insulin like growth factor 1 receptor (IGF1R) *in vivo*. We further found that attenuated XPO5-mediated export limitation of precursor miRNA is responsible for the downregulation of miR-378a-3p in SR cells. Our work implicates a causal role of the miR-378a-3p/IGF1R axis in sorafenib resistance and the therapeutic value of LXRα/ miR-378a-3p in HCC treatment.

## Materials and Methods

### Cell culture

Three human HCC cell lines (Huh7, HCCLM3 and SK-HEP-1) were purchased from the American Type Culture Collection (ATCC, Manassas, VA, USA). Cells were cultured according to the manufacturer's protocol and all cell lines were grown in DMEM supplied with 10% FBS at 37 °C with 5% CO_2_. We established SR cells *in vitro* by culturing the three cell lines in culture medium containing sorafenib for three months.

### miRNA sequencing

### HCC patients

A total of 100 randomly selected sorafenib- treated HCC patients at Sir Run-Run Shaw Hospital, Zhejiang University were included. Tumor and adjacent tumor tissues were collected. The study conformed to the principles of the Declaration of Helsinki and was approved by the Institutional Review Board of the Sir Run-Run Shaw Hospital. The clinicopathological information of the HCC patients included in this study is shown in Table S1.

### Quantitative real-time PCR (RT-qPCR) analysis

Total RNAs were extracted from tissue samples or cells using TRIzol (Invitrogen, USA) according to the manufacturer's instructions. Complementary DNA was synthesized from 1 µg of RNA using Hifair® II 1st Strand cDNA Synthesis SuperMix for qPCR (Yeasen, Shanghai, China) or All in-One miRNA qRT-PCR Detection Kit (GeneCopoeia, USA) when the product was used for miRNA detection. RT-qPCR was performed using Hieff UNICON® RT-qPCR SYBR Green Master Mix (Yeasen) or the All-in-One miRNA RT-qPCR Detection Kit when the product was used for miRNA detection. Assays were performed using the Roche LightCycler 480 or ABI Step One. Analysis was carried out using the ΔΔCt method. Primer sequences are listed in the supplementary data (Table S2 and Table S3).

### Oligonucleotide transfection

miRNA mimics (miR-378a-3p) and negative control miRNA were purchased from Ribobio (Guangzhou, China). miRNA mimics and negative control (50 nM) were transiently transfected into HCC cells using Lipofectamine 3000 reagent (Invitrogen, USA) or Lipofectamine RNAiMAX Reagent (Invitrogen) according to the manufacturer's protocol.

### Cell viability assay

Transiently transfected cells were seeded in a 96-well (0.5~1x10^4^/well) or 24-well plate (1~2x10^4^/ well) with three replicates. Cells were incubated with various concentrations of sorafenib for 48~72 h. Cell viability was then assessed by the Cell Counting Kit-8 (CCK-8) kit (Yeasen).

In other experiments, cells were cultured with different concentrations of sorafenib after cell adherence for more than 72 h. Data were collected by xCElligence RTCA MP (ACEA Biosciences, Hangzhou, China).

### Apoptosis assay

Cell apoptosis was detected by the PI/Annexin V-FITC apoptosis kit (MULTI SCIENCES, Hangzhou, China). Briefly, cells were seeded in 6-well plates (3~4x10^5^/well). Transfection was carried out as described previously. After incubation with sorafenib for 48~72 h, cells were harvested, washed once with PBS and resuspended in 500 µl 1X binding buffer. After the addition of 5 µl Annexin V-FITC and 10 µl PI, cells were incubated at room temperature for 5 min in the dark. The samples were analyzed with a BD LSRFortessa cell analyzer (BD Biosciences, USA). Data were analyzed by FlowJo software.

Cell apoptosis was also determined with TUNEL assay using the Direct TUNEL Apoptosis Assay Kit (Vazyme, Nanjing, China) according to the manufacturer's instruction. Briefly, after treatment, cells were washed twice by PBS and fixed by 1% formaldehyde in 4 °C for 20 min. Cells were then washed in PBS again and permeated by TrionX-100 for 5 min at room temperature. Cells were then incubated in 1X Equilibration buffer for 5 min at room temperature and the supernatant was removed. Cells were incubated in 50 µl TdT at room temperature for 60 min in the dark, followed by incubation in PI/RNase A Staining Buffer for another 30min. Cells were evaluated using a fluorescence microscope and 520 nm laser.

### Colony formation assays

Cells in single-cell suspension were plated in 6-well plates at a density of 1000 per well and cultured for 24 h. Cells were then treated or untreated with sorafenib or pharmacologic agents (NVP-ADW742, GW3965; from MCE, USA). The medium was replaced every 3 days with fresh medium containing the corresponding agents. The plates were further incubated for 14 days at 37 °C until colonies were visible. The colonies were fixed 15 min with 4% paraformaldehyde and stained with 0.1% crystal violet. The numbers of colonies were counted.

### Western blot analysis

Total proteins were extracted using RIPA lysis buffer (Beyotime, Shanghai, China) supplied with protease inhibitor cocktail (MCE) and phosphatase inhibitor cocktail (MCE). Proteins were separated by sodium dodecyl sulfate-polyacrylamide gel electrophoresis and transferred to a PVDF membrane (Millipore, USA). The membrane was blocked in skim milk (BD, USA) for 1 h at room temperature, followed by incubation overnight at 4°C with the appropriate antibody. The next day, after adequate washing in TBST and a 1-h incubation with the appropriate HPR-conjugated second antibody (Beyotime, Shanghai, China), the antigen-antibody complex on the membrane was detected with enhanced chemiluminescence regents (Fdbio Science, Hangzhou, China). All antibodies used in this study are listed in supplementary data (Table. S4).

### Immunohistochemistry

Tumor tissues from Sir Run-Run Shaw Hospital, Zhejiang University were used to examine IGF1R and Ki67 expression. Patients information is listed in the supplementary data (**Figure S3**) Immunohistochemistry was performed according to the previous report. Sections (3-µm-thick) were cut from routinely processed formalin-fixed, paraffin-embedded tissue blocks and subjected to immunohistochemistry staining with specific primary antibodies against IGF1R and Ki67. The slides were incubated with the primary antibody at 4 °C overnight. After washing with PBS, slides were processed using the GTvision immunohistochemistry kit according to the manufacturer's protocol.

### Luciferase reporter assay

The 3′-UTR of IGF1R containing the potential miR-378a-3p binding site was synthesized by TSINGKE Biological Technology (Beijing, China). The sequence was cloned into psiCHECK2 (Promega, USA). The 3′-UTR of IGF1R was inserted at the end of the Renilla luciferase gene and the firefly luciferase gene was used as an internal control. Cells transfected with miR-378a-3p mimics or control were then transfected with luciferase reporter plasmid for 48 h. The Promega Dual-Luciferase Reporter assay system (Promega, USA) was used to measure the activities of firefly and Renilla luciferase.

### RNA Fluorescence *In Situ* Hybridization (FISH)

We used the FISH kit from GenePharma (Shanghai, China) according to the manufacturer's protocol. The probe for pre-miR-378a was 5′-GTGCTATTTCTAGGTAACACACAG-3′.

### Cell fractionation

Cultured cells (10^7^) were collected, washed once in PBS, and placed on ice. Cells were resuspended in 500 μL ice-cold Cell Fractionation Buffer (Invitrogen, Shanghai, China) and incubated on ice 5-10 min. Samples were centrifuged and the supernatant (cytoplasmic fraction) was removed from the pellet (nuclear fraction). The pellet was mixed in 500 μL of ice-cold Cell Disruption Buffer (Invitrogen) and split the sample for RNA isolation.

### RNA Fluorescence *In Situ* Hybridization (FISH)

We used the FISH kit from GenePharma (Shanghai, China) according to the manufacturer's protocol. The probe for pre-miR-378a was 5′-GTGCTATTTCTAGGTAACACACAG-3′.

### Methylation specific (MS)-qPCR

We used the CpG Island Searcher web tool (www.cpgislands.com/) and identified one CpG island that spans the promoter region, the first exon, and part of the first intron of the XPO5 gene (-600 to +808). The MethPrimer program (www.urogene.org//methprimer) was then used to design methylation specific PCR primers within the identified CpG island region. To distinguish methylated and unmethylated DNA sequences, genomic DNA samples were bisulfite-treated using the DNA Methylation Kit (TIANGENBIOTECH, Beijing, China) according to the manufacturer's protocol. Upon bisulfite treatment, unmethylated cytosines are converted into uracil, whereas methylated cytosines remain unchanged. After conversion, the presence of methylation was determined by quantitative PCR using primers specific to the methylated or unmethylated sequence and the Hieff UNICON® RT-qPCR SYBR Green Master Mix (Yeasen), according to the manufacturer's protocol. The primer sequences used to detect unmethylated DNA were F: 5′-GGT GTG TTT AGT AAT GTA GTT GT-3′; R: 5′-CTA AAT AAA CAA AAC AAA AAA CAA A-3′; the primer sequences used to detect methylated DNA were F: 5′-GGC GCG TTT AGT AAT GTA GTC-3′; R: 5′-CTA AAT AAA CGA AAC GAA AAA CGA A-3′. The methylation index (MI) was calculated as MI = [1/ (1+2-(CTu-CTme)] × 100%, as previously described 19, in which CTu = the average cycle threshold obtained from duplicate qPCRs using the unmethylated primers, and CTme = the average cycle threshold obtained using the methylated primers.

### Animal studies

*In vivo* experiments were conducted using 4-week-old BALB/C nude mice (Shanghai SLAC laboratory Animal Co.,Ltd.). An orthotopic HCC model was constructed as described below. Briefly, mice were anesthetized by pentobarbital. An abdominal median incision was performed, and after liver exposure, a total of 5x10^8^ HCCLM3 cells were implanted into one lobe of the liver. The incision was closed using a suture of 5-0 silk. After tumor establishment, tumor tissues were harvested, cut into 16 pieces and re-implanted into the livers of 16 recipient 4-week-old BALB/C nude mice. When the tumors reached a length of ~2 mm, mice were randomized into four groups and assigned to receive vehicle, GW3965 (30 mg/kg), combination of sorafenib (30 mg/kg) with vehicle or combination of sorafenib (30 mg/kg) with GW3965 (30 mg/kg) every day. The model was monitored by IVIS every week. Mice were euthanized for analysis on day 30. Tumors were harvested and frozen in liquid nitrogen or fixed in 4% formalin immediately.

For the patient-derived xenograft (PDX), the liver tumor specimen of a 61-year-old male patient who had undergone hepatectomy at Sir Run-Run Shaw Hospital, Zhejiang University (Hangzhou, China) was obtained. Informed consent was obtained from the patient, and the study protocol was approved by an institutional review committee of Sir Run-Run Shaw Hospital, Zhejiang University. The specimen was directly implanted into the subcutaneous space of a NOD/SCID mouse (Shanghai SLAC laboratory Animal Co., Ltd.). After three months, the NOD/SCID mouse was euthanized and the engrafted tumor was implanted into four additional NOD/SCID mice in both flanks. When the tumors reached a volume of 500-800 mm^3^, tumors were collected, evenly cut into 6 pieces and re-implanted subcutaneously to 6 mice. When the tumors reached a length of ~3 mm, mice were randomized into two groups and assigned to receive either the combination of sorafenib (30 mg/kg) with vehicle or the combination of sorafenib (30 mg/kg) with GW3965 (30 mg/kg). Tumor volume was calculated using digital caliper measurements. Mice were euthanized for analysis on day 21. Tumors were harvested and frozen in liquid nitrogen or fixed in 4% formalin immediately. All animal studies were conducted according to the Association for the Assessment and Accreditation of Laboratory Animal Care and the Institutional Animal Care and Use Committee guidelines.

### Statistical analysis

Statistical analysis was performed using GraphPad Prism 7. Data are expressed as the mean ± standard error of the mean (SEM) from at least three independent experiments. Quantitative data between groups were compared using T test. Overall survival (OS) and disease free survival (DFS) curves were obtained by the Kaplan-Meier method, and differences were compared by log-rank test. A two-tailed P value of < 0.05 was considered statistically significant. For more detailed methods information please see Supporting Information.

## Results

### miR-378a-3p expression is reduced in SR cells and contributes to the development of sorafenib resistance

**Figure 1:**
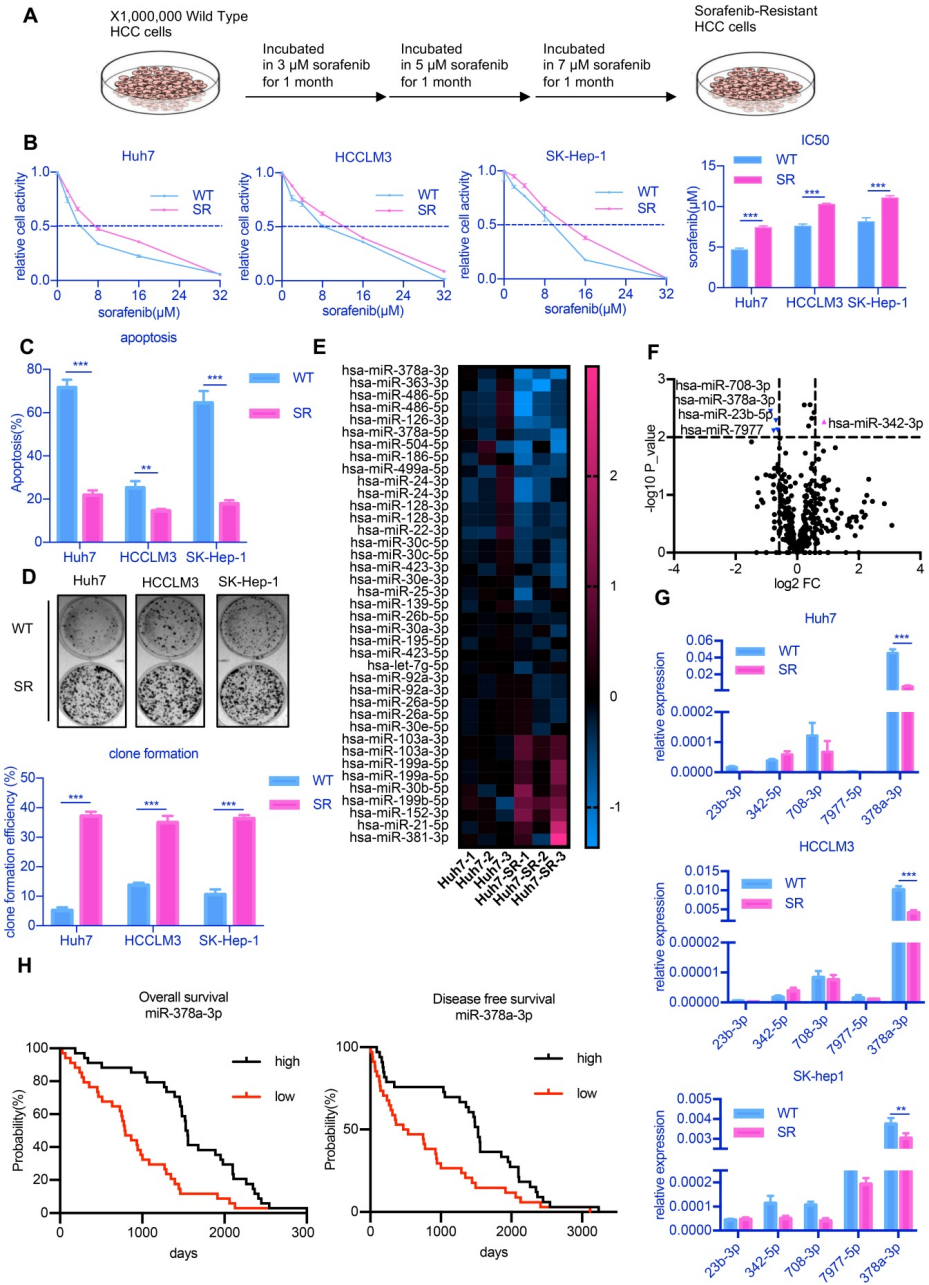
** miR-378-3p expression is reduced in sorafenib-resistant cells and may participate in resistance development. (A)** The process of the establishment of SR cells; **(B)** Cell viability was measured by CCK-8 assay at different concentration of sorafenib after 48 hours of dosing. The proliferation of resistant cells was all greater than that of their parental cells at different concentration of sorafenib; **(C)** Flow cytometry showed the apoptosis in sorafenib-resistant cells was less than parental cells; **(D)** Three sorafenib-resistant cells and parental cells were treated by sorafenib (10 μM). Colony-formation efficiency was carried out. Drug treatments were repeated every 3 days until colonies were visible; the emergency of sorafenib-resistant coloines was more than that of their parental cells; **(E)** miRNA sequencing of Huh7-SR and Huh7-WT showed a total of 39 miRNAs exhibited significantly different expression between the two groups; **(F)**Five miRNAs (miR-23b-3p, miR-342-3p, miR-708-3p, miR-797-3p, and miR-378a-3p, fold change >1.5 and p<0.01) were significantly reduced in Huh7-SR cells; **(G)** RT-qPCR evaluated the expression of the five candidates in all three resistant cell lines. The results confirmed that these five miRNAs were all downregulated in sorafenib-resistant cells. **(H)** HCC prognosis data obtained from KM-plotter showed patients with higher miR-378a-3p levels in cancer tissue had significantly better overall (HR=0.42; 95%CI: 0.25 to 0.71; P=2.0e-0.4) and disease-free survival (HR=0.53; 95%CI: 0.32 to 0.88; P=0.0069).

To examine the molecular mechanisms underlying sorafenib resistance in HCC, we established SR HCC cell lines by culturing three HCC cell lines in culture medium containing increasing doses of sorafenib over 3 months 20 (**Figure 1A**). IC50, the half maximal inhibitory concentration, represents the concentration of a drug that is required for 50% inhibition of things. An increase in IC50 means that cells are more resistant to drugs. The three drug-resistant cell lines were designated as Huh7-SR, LM3-SR and SK-Hep-1-SR and IC50 value of these cells were significantly increased (**Figure 1B**). The percentages of apoptotic cells in the three drug-resistant strains were all lower than that of the parental cell lines after treatment with 10 µM sorafenib (**Figure 1C**). Sorafenib was also unable to inhibit colony growth in the SR cell lines (**Figure 1D**).

Previous studies showed that miRNAs represent novel therapeutic targets in HCC 21. Thus, to explore potential miRNA candidates that may be involved in sorafenib resistance development, we performed miRNA sequencing on Huh7-SR and Huh7 cells, as these cells showed the greatest difference in sensitivity to sorafenib among all three cell lines. The expression of five candidate miRNAs (miR-23b-5p, miR-342-3p, miR-378a-3p, miR-708-3p, miR-7977-5p, fold change >1.5 and p<0.01) identified by miRNA sequencing (**Figure 1E and Figure 1F**) were confirmed through RT-qPCR analysis. We observed consistent low expression of miR-378a-3p in all three SR HCC cell lines (**Figure 1G**).

To determine the significance of miR-378a-3p in the clinical setting, we examined miR-378a-3p levels in the samples of 100 randomly selected sorafenib-treated HCC patients and found that patients with significantly higher levels of miR-378-3p had significantly better OS (HR=0.42; 95%CI: 0.25 to 0.71; p=2.0e-4) and better DFS (HR=0.53; 95%CI: 0.32 to 0.88; p=0.0069) (**Figure 1H**).

### miR-378a-3p overexpression sensitizes HCC response to sorafenib

**Figure 2:**
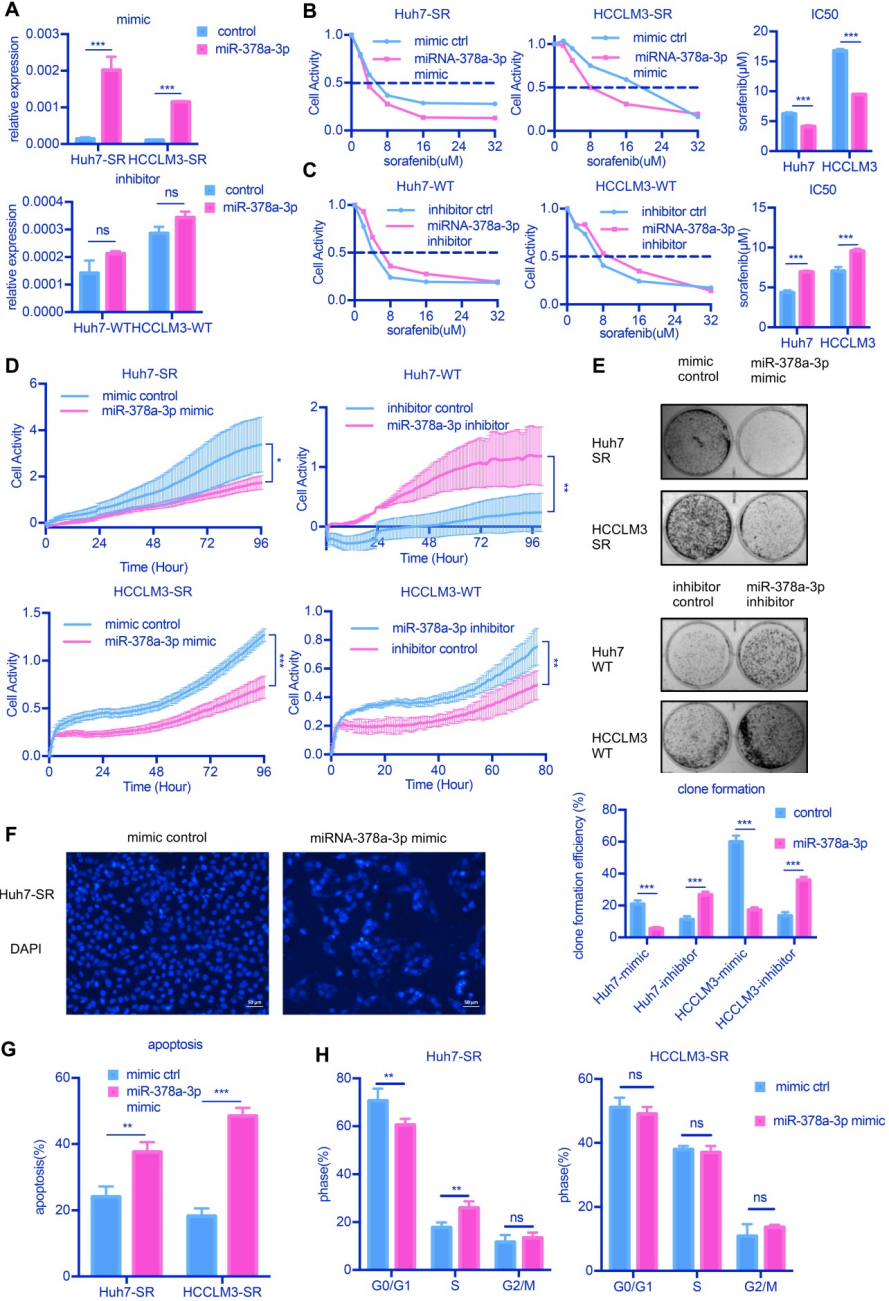
** miR-378a-3p could sensitize cellular response to sorafenib. (A)** The expression of miR-378a-3p in Huh7 cells transfected with miR-378a-3p mimic or miR-378a-3p inhibitor; **(B,C)** The proliferation of Huh-SR cells and HCCLM3-SR cells was all greater than that transfected with miR-378a-3p mimic at different concentration of sorafenib; Meanwhile, the proliferation of Huh7-WT cells and HCCLM3-WT cells which transfected with miR-378a-3p inhibitor was all greater than that Huh7-WT cells at different concentration of sorafenib; **(D)** Real-time cellular analysis showed Huh7-SR cells and HCCLM3-SR cells which transfected with miRNA-378a-3p mimics were more sensitive to sorafenib than control cells; **(E)** Colony-formation efficiency showed the emergency of sorafenib-resistant colonies which transfected with miR-378a-3p mimics was less than that of their parental cells; **(F)** Fluorescence microscopy image post-miR-378a-3p transfection and incubation with sorafenib for 48 h. **(G)** Flow cytometry showed miR-378a- 3p transfection could induce apoptosis in Huh-SR cells and HCCLM3-SR cells; **(H)** Flow cytometry showed miR-378a- 3p transfection did not significantly affect cell-cycle distribution in Huh-SR cells and HCCLM3-SR cells.

To explore the role of miR-378a-3p in sorafenib resistance, we examined the effects of miR-378a-3p over-expression in response to sorafenib on the proliferation, apoptosis and clonal formation abilities of HCC cells *in vitro*. SR cells, which had lower miR-378a-3p levels, were transfected with miRNA mimics, while parental cells, which had higher miR-378a-3p levels, were transfected with miRNA inhibitor; and the expression of miR-378a-3p was verified by RT-qPCR (**Figure 2A**). The inhibitor of miRNA could not make the miRNA degradation, so qPCR did not show any difference.

Overexpression of miR-378a-3p deceased the IC50 values of sorafenib obviously (**Figure 2B**), while miR-378a-3p inhibitor treatment in parental cells increased the IC50 values of sorafenib compared with controls (**Figure 2C**). Using real-time cellular analysis, we found Huh7-SR cells and HCCLM3-SR cells which transfected with miRNA-378a-3p mimics were more sensitive to sorafenib than control cells, in the meanwhile, miR-378a-3p inhibitor had the opposite effect (**Figure 2D**). Colony formation assay also showed that upregulation of miR-378a-3p in SR cell lines inhibited colony growth (**Figure 2E**).

We next explored whether the antiproliferation activity induced by miR-378a-3p and sorafenib in SR cells was due to apoptosis. We found that increasing miR-378a-3p levels by transfection of mimics could induce cell apoptosis as observed nuclear morphological changes (**Figure 2F** and**Figure S1A**). The combined treatment of miR-378a-3p mimics and sorafenib in SR cells resulted in significantly higher apoptotic rates (from 24.08% to 37.64%, P<0.001 and from 18.29% to 48.50%, P<0.001 in Huh7-SR and HCCLM3-SR cells, respectively) compared with sorafenib treatment alone (**Figure 2G**). However, the combination treatment did not produce significant changes in the cell cycle compared with sorafenib alone (**Figure 2H**). Together these results indicate that miR-378a-3p overexpression sensitizes HCC cells to sorafenib by induction of apoptosis.

### IGF1R is a novel direct target of miR-378a-3p in sorafenib resistance

**Figure 3:**
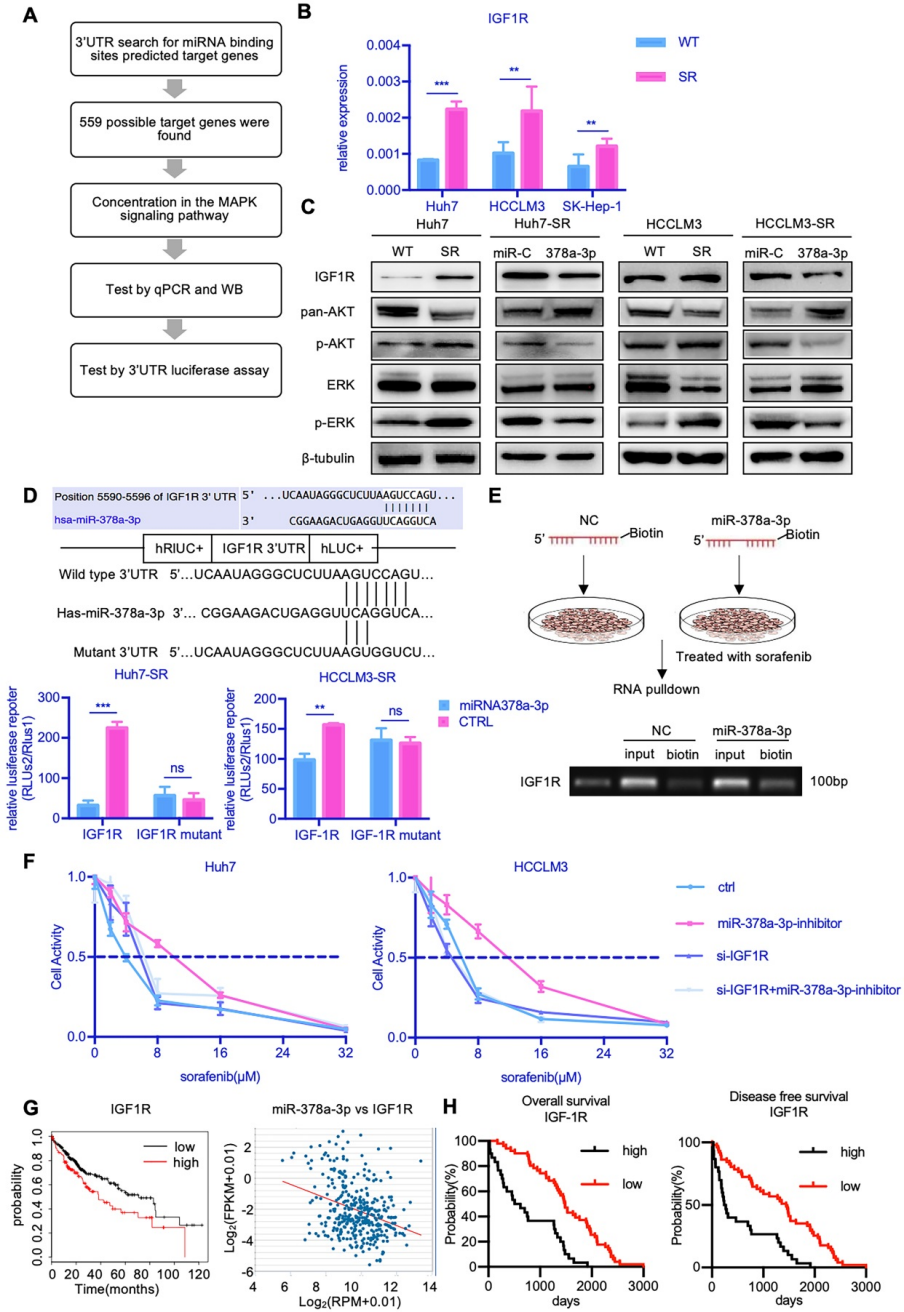
** miR-378a-3p participated in sorafenib resistance most likely by targeting IGF1R. (A)** The process of research for target genes; **(B)** RT-qPCR revealed mRNA levels of IGF1R were significantly higher in Huh7-SR, HCCLM3-SR and SK-Hep-1-SR cells compared with their parental lines; **(C)**WB showed IGF1R were significantly upregulated in resistant cell lines along with their common downstream target pERK/p-AKT; **(D)** The conserved miR-378a-3p cognate site in 3′-UTR of IGF-1R;Verification of the conserved miR-122 cognate site in 3′-UTR of IGF-1R. Luciferase activity driven by 3′-UTR of IGF-1R was inhibited by ectopic expression of miR-122. Huh7 cells were co-transfected with firefly luciferase-3′-UTR-IGF-1Ror mutant 3′-UTR of IGF-1R with the miR-378a-3p complementary site deleted and miR-122 mimics or control RNA (100 nM) along with a control plasmid; Reduction of luciferase activity driven by IGF-1R-3′UTR was observed (left panel). The IGF-1R-3′UTR-Mutant construct failed to suppress the luciferase activity (right panel) in Huh7 and HCCLM3; **(E)** The process of RNA pulldown; RNA pulldown assay revealed miR-378a-3p could bind to both sites in IGF1R 3'UTR; **(F)** IGF1R knockdown significantly blocked the miR-378a-3p inhibitor-mediated sorafenib resistance of HCC cells; **(G)** HCC prognosis data in TCGA obtained from KM-plotter showed patients with lower IGF1R levels in cancer tissue had significantly better overall (HR=1.58; 95%CI: 1.11 to 2.25; p=0.01); there was negative correlation between miR-378a and IGF1R in the TCGA LIHC dataset (R=-0.330, p=7.53e-11); **(H)** HCC prognosis data obtained from KM-plotter showed patients with lower IGF1R levels in cancer tissue had significantly better overall (HR=4.96; 95%CI: 2.69 to 9.14; p<0.0001) and disease-free survival (HR=2.63; 95%CI: 1.49 to 4.61; p<0.0001).

To explore the mechanism by which miR-378a-3p mediates sorafenib resistance in HCC cells, we searched for candidate downstream targets of miR-378a-3p using publicly available databases (i.e., miRwalk, miRanda, and Targetscan databases). Based on the database findings and literature review, we selected IGF1R for further experimental validation (**Figure 3A**).

RT-qPCR showed that the mRNA expression of IGF1R was markedly increased in all three SR cells (**Figure 3B**). We also found that the protein level of IGF1R were upregulated in Huh7-SR cells compared with Huh7-WT cells (**Figure 3C**). Furthermore, the levels of IGF1R protein expression were decreased in Huh7-SR cells transfected with miR-378a-3p mimics. Together, these findings indicated that IGF1R may be a downstream target of miR-378a-3p.

To confirm this hypothesis, we carried out Targetscan *in silico* analysis and identified a potential miR-378a-3p binding site in the 3′-UTR of the IGF1R mRNA (**Figure 3D**). We thus constructed luciferase reporter plasmids containing either the wild-type 3′-UTR or mutant 3′-UTR of IGF1R which lost the potential of binding to miR-378a-3p. While miR-378a-3p significantly suppressed the luciferase activity of the reporter vector containing the wild-type 3′-UTR in SR cells, it had no impact on the reporter vector containing the mutant 3′-UTR (**Figure 3D**). In addition, we synthesized biotinylated miR-378a-3p, which had a biotin tagged to its 3' end. After miR-378a-3p-biotin transfection, RNA pulldown was performed and the result revealed that this oligo could bind to target site of IGF1R 3'UTR (**Figure 3E**). This experiment verified the physical binding capacity between miR-378a-3p and 3'UTR of IGF1R.

IGF1R activates both the PI3K/AKT pathway and the Ras/Raf/MAP kinase pathway. Given that these pathways play important roles in mediating cell survival, we examined the effect of IGF-1R activation on the expression of components of the MAPK and AKT signaling pathways. Notably, Huh7-SR cells expressed high levels of IGF1R, as well as increased phospho-AKT1 and phospho-ERK1/2, which represents signaling activation (**Figure 3C**). Furthermore, upon transfection of Huh7-SR cells with miR-378a-3p mimics, the levels of IGF1R, phospho-AKT1, and phospho-ERK1/2 were reduced. To further confirmed miR-378a-3p/IFG1R axis, rescue experiments has been performed. As shown in **Figure 3F**, IGF1R knockdown significantly blocked the miR-378a-3p inhibitor mediated sorafenib resistance of HCC cells. So, IGF1R knockdown could block the function of miR-378a-3p inhibitor. Meanwhile, the treatment of IGF1R inhibitor, NVP-AEW742, combined with sorafenib dramatically suppressed the proliferation of SR cells, and upon treatment of NVP-AEW742 combined with miR‐378a-3p-inhibitor, the proliferation of cells was partially rescued **(Figure S1B)**. These data convincingly demonstrate that miR-378a-3p suppresses HCC sorafenib resistance as an inhibitor of IGF1R to eliminate the IGF1R resistant effect through miR-378a-3p/IGF1R axis.

Data from TCGA also suggested clinical relevance that patients with higher levels of IGF1R had worse prognosis. Moreover, there was negative correlation between miR-378a-3p and IGF1R in the TCGA LIHC dataset (**Figure 3G**). Furthermore, sorafenib-treated HCC patients with negative IGF1R expression showed significantly better OS (HR=4.96; 95%CI: 2.69 to 9.14; P<0.0001) and DFS (HR=2.63; 95%CI: 1.49 to 4.61; P<0.0001) than those with high IGF1R expression (**Figure 3H**).

### Attenuated XPO5-mediated export of pre-miR-378a in SR HCC cells

**Figure 4:**
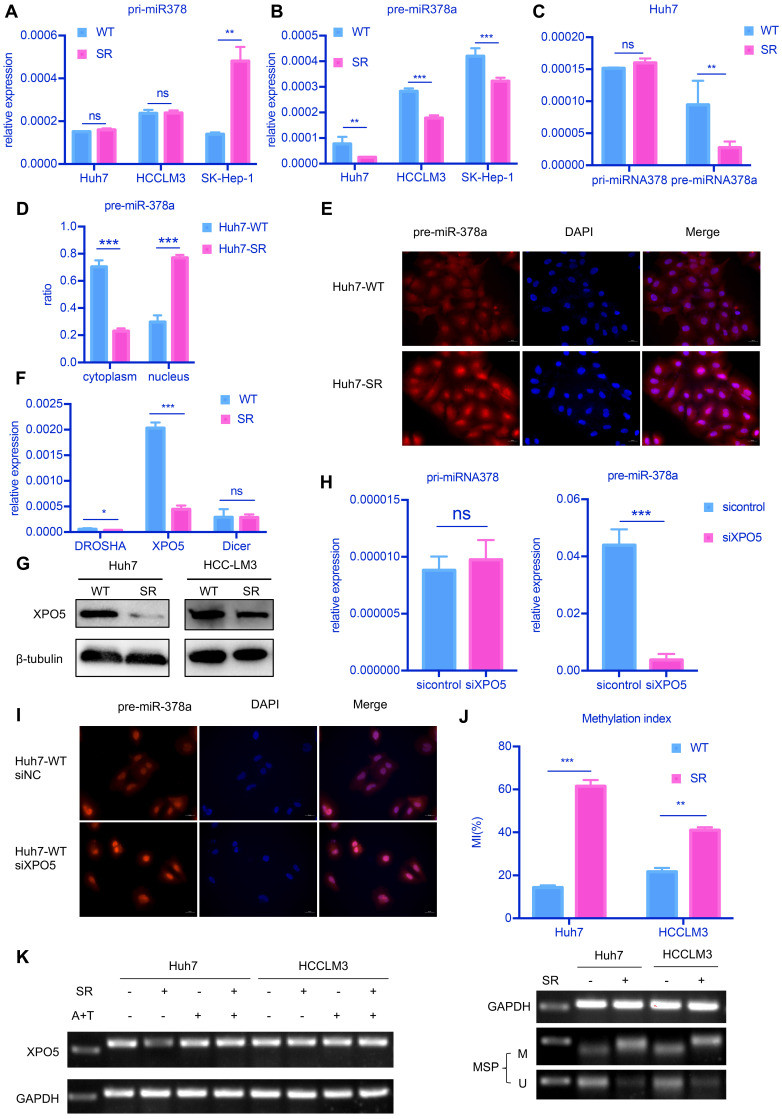
** attenuated XPO5-mediated export of pre-miR-378a. (A)** RT-qPCR revealed the levels of the pri-miR-378a were no different in Huh7-SR, HCCLM3-SR and SK-Hep-1-SR cells compared with their parental lines. **(B)** RT-qPCR revealed the levels of pre-miR-378a were lower in Huh7-SR, HCCLM3-SR and SK-Hep-1-SR cells compared with their parental lines. **(C)**There was a obvious uncouple of pri-miR-378 and pre-miR-378a in Huh7-SR cells compared with Huh7-WT cells; **(D)** RT-qPCR revealed the content of precursors in Huh7-SR cytoplasm decreased significantly; **(E)** FISH showed the pre-miR-378a in Huh7-SR was trapped in the nucleus; **(F)** RT-qPCR revealed the levels of XPO5 was lower in Huh7-SR compared with Huh7-WT. **(G)** WB showed XPO5 were significantly down regulated in Huh7-SR and HCCLM3-SR. **(H)** After knockdown XPO5 in Huh7-WT, RT-qPCR revealed the levels of pre-miR-378a was reduced. **(I)** After knockdown XPO5 in Huh7-WT, the pre-miR-378a was also trapped in the nucleus. **(J)** DNA methylation was detected in Huh7-SR cells, Huh7-WT cells HCCLM3-SR cells and HCCLM3-WT cells by MSP; **(K)** RT-PCR showed that XPO5 mRNA expression was restored after treatment with demethylation agent 5-aza-2′-deoxycytidine (5-Aza) in Huh7-SR cells, Huh7-WT cells HCCLM3-SR cells and HCCLM3-WT cells.

To explore the cause of the decrease of miR-378a-3p in SR HCC cells, we examined levels of primary-miR-378 (pri-miR-378) and precursor-miR-378a (pre-miR-378a) in the HCC lines by RT-qPCR (**Figure 4A** and **Figure 4B**). We detected a significant uncoupling of pri-miR-378 and pre-miR-378a in SR cells (**Figure 4C**). The expression of pre-miR-378a was much lower in SR cells compared with parental cells. This suggested that there may be some factors causing this phenomenon in the process from pri-miR-378 to pre-miR-378a.

We further explored the distribution of pre-miR-378a in nuclear and cytoplasmic fractions of SR cells and parental cells. We found that the proportion of pre-miR-378a was much lower in cytoplasmic fractions of Huh7-SR cells than in Huh7-WT cells by cell fractionation qPCR (**Figure 4D**) and RNA FISH also showed that pre-miR-378a was present at high levels in cell nuclei of Huh7-SR cells (**Figure 4E**).

To further investigate the reason for decreased miR-378a-3p in SR HCC cells, we examined the mRNA levels of key proteins involved in the production of pre-miRNA, including DROSHA, DICER1 and Exportin5 (XPO5). XPO5 mRNA and protein levels were significantly decreased in Huh7-SR cells compared with parental cells (**Figure 4F** and **Figure 4G**). Knockdown of XPO5 resulted in decreased expression of pre-miR-378a and had no effect on pri-miR-378 (**Figure 4H** and**Figure S1C**). RNA FISH also showed that pre-miR-378a was more strongly detected in the nucleus of XPO5 knockdown cells (**Figure 4I**). Meanwhile when XPO5 was partially knocked down, cell proliferation and viability has been increased (**Figure S1D** and**Figure S1E**). But When XPO5 was further knocked down, cell viability went down instead of increasing (**Figure S1D** and**Figure S1E**).

Next, we explored the mechanism by which XPO5 mRNA was downregulated in SR cells. We identified a CpG island that spans the promoter region, the first exon, and part of the first intron of the XPO5 gene (-600 to +808). We thus evaluated the methylation status of the XPO5 promoter by MS-qPCR. Methylation of this region was detected in the two SR cell lines but not in the parental cells (**Figure 4K**). To further determine whether promoter methylation was involved in the downregulation of XPO5, the two SR cell lines were treated with 5-Aza and TSA. Restored expression of XPO5 was observed in SR cells upon 5-Aza and TSA treatment (**Figure 4L**).

### LXRα agonist potentiates sorafenib sensitivity by facilitating transcription of miR-378

**Figure 5:**
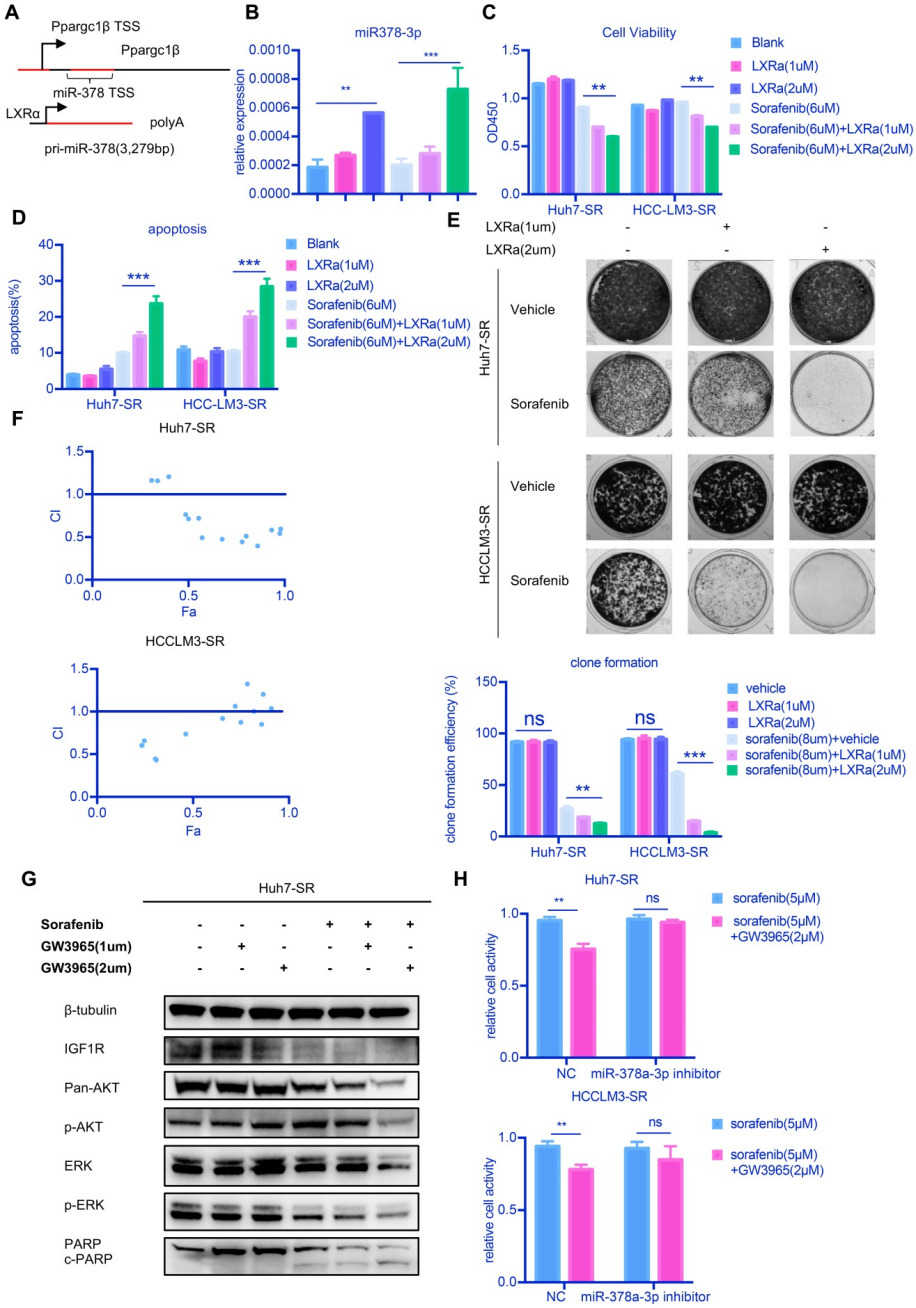
** LXRα agonist could increase miR-378a-3p and potentiated sorafenib sensitivity. (A)** miR-378 possesses its own transcription machinery independent of its host gene; **(B)** LXRα agonist could increase the level of miR-378a-3p in Huh7-SR. **(C)** GW3965 could increase the effect of sorafenib in Huh7-SR and HCCLM3-SR; **(D)** Flow cytometry showed the combination of sorafenib and GW3965 could induce apoptosis in Huh7-SR and HCCLM3-SR; **(E)** Colony-formation efficiency showed the emergency of Huh7-SR coloines which treated with sorafenib and GW3965 was less than that treated with sorafenib alone; **(F)** Cells were seeded in 24-well plates (5X10^3^cells/well) and incubated overnight for attachment, and were then treated with indicated doses of GW3965 and sorafenib for 48 hr. The impact of Sorafenib, GW3965 and the combination therapy on cell viability in Huh7-SR and HCCLM3-SR cells were determined by CCK8 assay. The CI values of each dose were calculated by the CompuSyn software and CI < 1 indicated synergism; **(G)** WB showed IGF1R were upregulated in combination along with their common downstream target pERK/p-AKT and combination treatment also could induce apoptosis; **(H)** Transfected with miR-378a-3p inhibitor could partially rescue the effect of combination therapy in Huh7-SR and HCCLM3-SR.

Our results thus far suggest that upregulation of miR-378a-3p in HCC and/or enhancing its nuclear export may be of therapeutic benefit to resensitize HCC cells to sorafenib. However, there is currently a lack of targeted drugs for XPO5. To increase the clinical application of miR-378a-3p, we used an agonist for LXRα (GW3965), a transcription factor that regulates miR-378 expression 14 (**Figure 5A**). We confirmed that GW3965 increased miR‐378a-3p expression in Huh7-SR cells (**Figure 5B**). Combined treatment of LXRα and sorafenib significantly inhibited the growth of Huh7-SR cells and HCCLM3-SR cells (**Figure 5C**). Treated with GW3965 alone did not affect the proliferation of HCC cells (**Figure 5C**). The combination therapy also dramatically increased the percentage of apoptotic population to 23.7% in Huh7-SR cells compared with sorafenib therapy alone (9.9%) (**Figure 5D**) and the combination therapy nearly eliminated the emergence of sorafenib-resistant cells (**Figure 5E**). In the meanwhile, GW3965 treatment alone did not induce apoptosis and affect the clonal formation in SR cells (**Figure 5D** and** Figure 5E)**. The CI values of each dose were calculated by the CompuSyn software and the results suggested that GW3965 exhibited a synergistic effect in combination with sorafenib to suppress Huh7-SR cells and HCCLM3-SR cells, indicating that this synergistic effect was not a cell line-specific effect (**Figure 5F**). The combined treatment also induced a decline of IGF1R protein and inhibition of AKT1 and ERK1/2 phosphorylation compared with sorafenib alone (**Figure 5G**) and GW3965 treatment alone did not show any difference. Together these results demonstrated that GW3965 could increase the killing effect of sorafenib in resistant cells.

To further confirm our hypothesis, we examined whether miR‐378a-3p mediates the sensitization effect of LXRα on HCC cells to sorafenib. Huh7-SR and HCCLM3-SR cells incubated with sorafenib were treated with either GW3965 or a combination of miR‐378a-3p inhibitor and GW3965 and then cells were evaluated by CCK8 assays (**Figure 5H**). The antiproliferation effect of the combination therapy was weakened in Huh7-SR and HCCLM3-SR cells transfected with miR-378a-3p-inhibitor. This indicated that miR-378a-3p-inhibitor could offset the sensitization effect of LXRα on sorafenib by antagonizing GW3965-mediated elevation of miR‐378a-3p. This mean GW3965 was working through miR-378a-3p to sensitive HCC cells to sorafenib.

### The combination of LXRα agonist and sorafenib suppresses HCC proliferation *in vivo*

**Figure 6:**
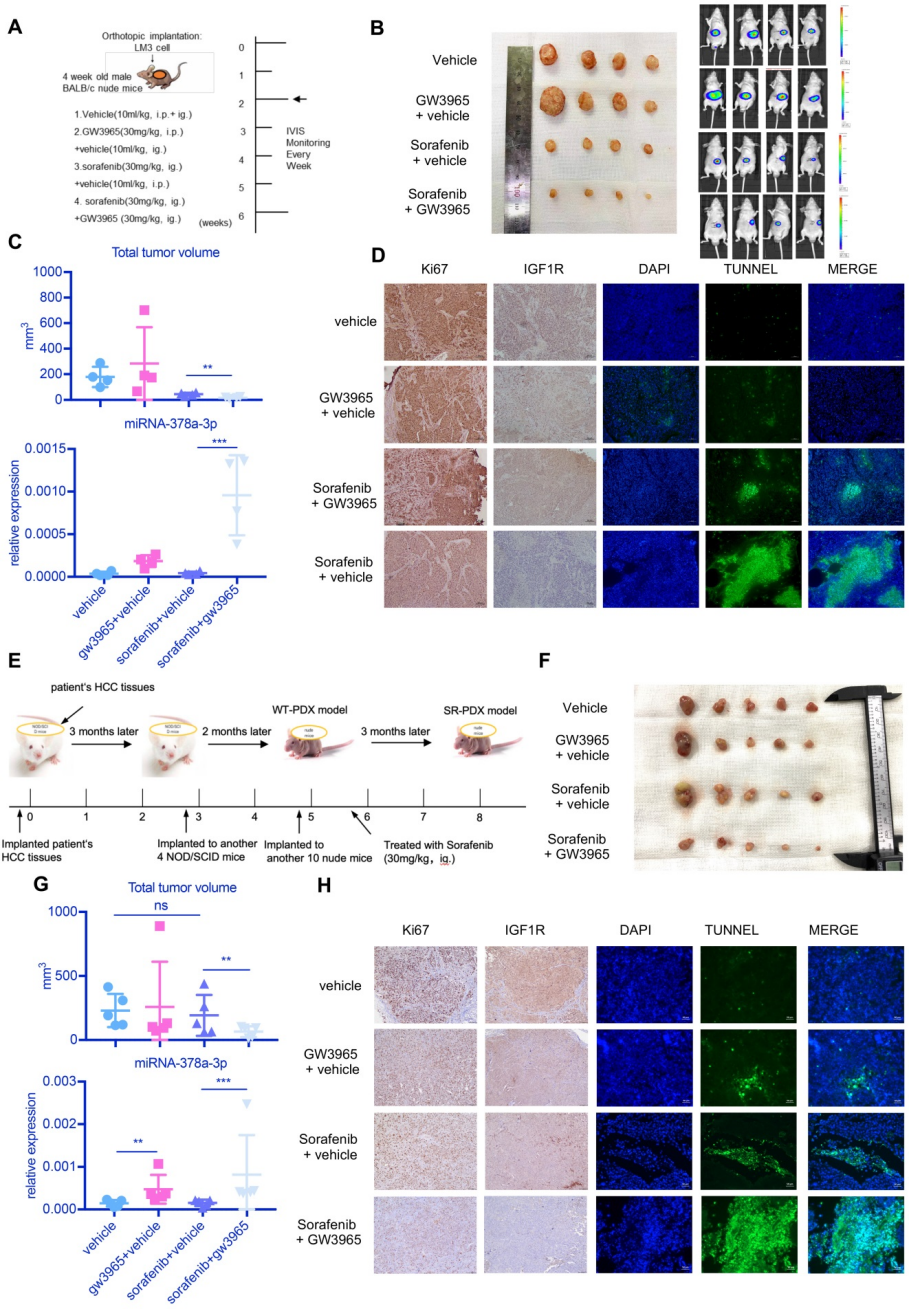
***in vivo* experiment showed the combination sorafenib and GW3965 could suppress HCC proliferation. (A)** Schematic representation of the *in vivo* model timeline; The IVIS monitor showed combination therapy could suppress HCCLM3 proliferation **(B)** gross view of tumors from four groups; **(C)** The total volume of tumors from four groups; miRNA levels of four groups were verified using RT-qPCR; **(D)** Immunohistochemistry showed combination therapy could suppress HCC proliferation and IGF1R level *in vivo*; Meanwhile, TUNEL assay showed combination therapy induced more apoptosis; **(E)** Schematic representation of the SR-PDX model timeline; **(F)** gross view of tumors from four groups; Liver view of tumors from four groups; **(G)** The total volume of tumors from four groups; miRNA levels of four groups were verified using RT-qPCR; **(H)** Immunohistochemistry showed combination therapy could suppress HCC proliferation, IGF1R level and TUNEL assay in SR-PDX model also showed combination therapy induced more apoptosis.

To examine the antitumor effects of the combination treatment against HCC *in vivo*, we used a subcutaneous mouse xenograft model (**Figure 6A**). Combined therapy of sorafenib with GW3965 demonstrated a significant inhibitory effect on tumor growth compared with sorafenib alone (**Figure 6A** and **Figure 6B**). Figure 6C showed the combination treatment could increase miR-378a-3p and suppress tumor proliferation. In addition, the ALT level and weight of mice showed no changes (**Figure S2A**), indicating the combination therapy did not result in more severe side effects than sorafenib therapy alone (**Figure 6C**). Meanwhile, IHC staining from these mice HCC tumors revealed similar patterns of change as those we found *in vitro* in IGF1R and apoptosis (**Figure 6D**).

We further established a PDX model derived from a 61-year-old male patient with HCC (**Figure S2B**). We also found that the combination treatment had better effect on reducing tumor size than sorafenib alone (**Figure S2C**) and the combination treatment resulted in more apoptosis than sorafenib treatment alone (**Figure S2D**). In two of the three mice treated with sorafenib alone, there were many liver metastases (**Figure S2D**). HE staining can also confirm this (**Figure S2D**). These results indicated that GW3965 combined with sorafenib possibly could suppress the invasiveness of HCC.

As shown in **Figure 6E**, we established sorafenib-resistant (SR) PDX model by treating PDX mice with sorafenib (30mg/kg, ig.) over 2 months. Then we examined the antitumor effects of the combination treatment against HCC in this SR-PDX model. Sorafenib treatment alone was not able to inhibit the proliferation of tumor, but combined therapy of sorafenib with GW3965 demonstrated a significant inhibitory effect on tumor growth (**Figure 6F**). Figure 6G showed the combination treatment could increase miR-378a-3p and suppress tumor proliferation. Meanwhile, IHC staining from these mice HCC tumors revealed similar patterns of change as those we found *in vitro* in IGF1R and apoptosis (**Figure 6H**).

Together these results revealed that the LXR agonist could be a promising novel combination therapy with sorafenib to overcome sorafenib resistance in sorafenib-tolerant HCC patients.

## Discussion

Sorafenib is an effective therapy for advanced HCC and affects the PI3K/Akt 22 and JAK-STAT signaling pathways^23^, hypoxia-inducible pathways 24, epithelial to mesenchymal transition events 25, 26, Mapk14-dependent activation of MEK-ERK 27 , GSK-3β 28 and Atf2 signaling 29, 30. The broad spectrum of activity of sorafenib suggests a complex plethora of events that may contribute to the development of acquired resistance 3. In the sorafenib-resistant mouse model, the mechanisms that ultimately lead to upregulation of IGF1R could involve, among others, miRNA or epigenetic regulation 17. On the basis of miR‑378a enhancing the sensitivity of liver cancer to sorafenib by targeting VEGFR, PDGFRβ and c‑Raf^31^, we further confirmed that the expression of miR-378a-3p was decreased in sorafenib-resistant HCC cells and identified IGF1R as the target gene of miR-378a-3p.

The biogenesis of miRNAs is tightly controlled at multiple steps, such as transcription of miRNA genes 32, 33, processing by Drosha 34 and Dicer 8, and transportation of pre-miRNAs from the nucleus to the cytoplasm by XPO5/RANGTP 35, 36. Nuclear export of pre-miRNA by XPO5 is a critical step for miRNA biogenesis 37, 38, and XPO5 expression requires accurate regulation to maintain its normal function under physiological conditions. Recent studies showed that genetic and epigenetic changes in the XPO5 gene as well as abnormal expression or modifications of XPO5 protein are associated with certain tumors 39. These alterations in XPO5 may lead to abnormal expression of mature miRNAs. Epigenetic modifications including DNA methylation and histone modification are altered during tumorigenesis and may trigger resistance to immune surveillance, chemotherapy, and targeted drugs 40, 41. DNA hypermethylation at CpG islands in gene promoter usually leads to gene silencing. A previous study reported decreased methylation in the CpG island in the XPO5 gene (-600 to 808), which may explain the upregulation of XPO5 expression in breast cancer 39. We also found methylation of this region in the SR cell lines and treatment of resistant cells with 5-Aza and TSA led to reactivation of XPO5 expression 42. Whether RNA modification or histone acetylation could also affect XPO5 gene expression in SR cells remains elusive. But recently, XPO5 is found to act as an oncogene in colorectal cancer due to its high expression in CRC and anti-tumor effect after XPO5 knockdown 43. Therefore, there may be some risks in targeting XPO5 for cancer treatment.

Nuclear receptors are master regulators of transcriptional programs that integrate the homeostatic control of almost all biological processes 44. Their direct mode of ligand regulation and genome interaction is at the core of modern pharmacology 45. The liver X receptors (LXRs) are emerging drug targets within the nuclear receptor family. LXRs are nuclear oxysterol receptors and physiological regulators of lipid and cholesterol metabolism that also act in an anti-inflammatory way. Because LXRs control diverse pathways in development, reproduction, metabolism, immunity and inflammation, they have potential as therapeutic targets for diseases as diverse as lipid disorders, atherosclerosis, chronic inflammation, autoimmunity, neurodegenerative diseases and cancer 46. Recent insights into LXR signaling suggest future targeting strategies aiming at increasing LXR subtype and pathway selectivity. Thus, LXRs are new potential drug targets in cancer treatment. Here we showed that LXRα functions as an activator of miR‐378 transcription in SR HCC cells and could reverse sorafenib resistance in these cells. Moreover, the combination of GW3965 and sorafenib also suppressed IGF1R and subsequently inhibited the activation of ERK/PI3K signaling. Furthermore, combination treatment in the CDX and PDX model further showed that GW3965 inhibited tumor proliferation with sorafenib treatment.

In conclusion, here we demonstrated that miR-378a-3p was involved in the sorafenib-resistance of HCC cells via downregulation of IGF1R. Moreover, we identified novel mechanisms for miRNA downregulation in SR cells and revealed that XPO5-mediated miRNA export limitation plays a role in the development of sorafenib resistance. Our results indicate that LXRα may be an effective target for anti-sorafenib-resistance therapies for liver cancer patients.

## Supplementary Material

Supplementary figures and tables.
