# Supplementary material for: LXR activation potentiates sorafenib sensitivity in HCC by activating microRNA-378a transcription
Source: Theranostics. 2020 Jul 11;10(19):8834–50. doi: 10.7150/thno.45158 (PMC7392029; doi:10.7150/thno.45158)
Supplement: Supplementary file 1 — Supplementary figures and tables. [file thnov10p8834s1.pdf]

Supplementary Table 1. Clinical and Pathological Features of the 100 HCC Cases

	No. of cases
Gender	
Male	92
Female	8
Age	
Median(range)	47.2(16.0-73.0)
< 70	99
> 70	1
Serology	
HBV	79
HCV	0
Cirrhosis	
No	54
Yes	46
Tumor size	
< 5 cm	47
> 5 cm	53
Tumor differentiation	
Moderately	86
Poorly	14

Supplementary Table 2. List of the Primers Used for Real-time Quantitative PCR

Gene	Name	Sense	Antisense
GAPDH	Glyceraldehyde 3-phosphatase dehydrogenase	acccactcctccacctttga	tccaccaccctgttgctgta
IGF1R	Insulin like growth factor 1 receptor	agactgaacctctgttgccag	tggggtcagggagaaatcct
DROSHA	Drosha ribonuclease III	gaccaagtattcagcaagcgg	agtctagggtcacaatctggg
XPO5	Exportin 5	gcattgctgggtgcagattc	ggctccccaagtcatctgag
DICER	Dicer	tcgagcacatgagaccaacc	atggtcatccagttcgccaat
pri-miR-378	Primer-microRNA-378	ggtggtgccgcaagagaatc	tgcaggaacaaccagaacat ct
U6	U6 small nuclear RNA	ctcgcttcggcagcaca	aacgcttcacgaatttgcgt

The primers were designed using Primer3 v.0.4.0 (http://bioinfo.ut.ee/primer3-0.4.0/primer3) and purchased from Tsingke.

Supplementary Table 3. List of the Primers Used for microRNA Real-time Quantitative PCR

Gene	Sense
microRNA-378a-3p	actggacttggagtcagaaggc
microRNA-342-3p	tctcacacagaaatcgcaccc
microRNA-23b-5p	tgggttcctggcatgctgat
microRNA-7977	ttcccagccaacgcacca
microRNA-708-3p	gggcccaactagactgtgagc
Precusor-microRNA-378a-3p	tgagggctcctgactccagg
5s	gggaataccgggtgctgtaggct

The primers were designed designed on the basis of a study by Shi and collaborators (Shi et al., 2011) and purchased from Tsingke.

Supplementary Table 4 Antibodies used in the paper

Antibody	Catalogue number	Company
IGF1R	#9750	CST
Cleaved PARP (Asp214)	#5625	CST
ERK1/2	#4695	CST
pERK1/2(pT202/pY204)	#4370T	CST
Akt (pan)	#4691	CST
Phospho-Akt (Ser473)	# 4060	CST
Exportin-5	ab129006	Abcam
β-Tubulin	AT809	Beyotime

Supplemental Figure and Figure Legends

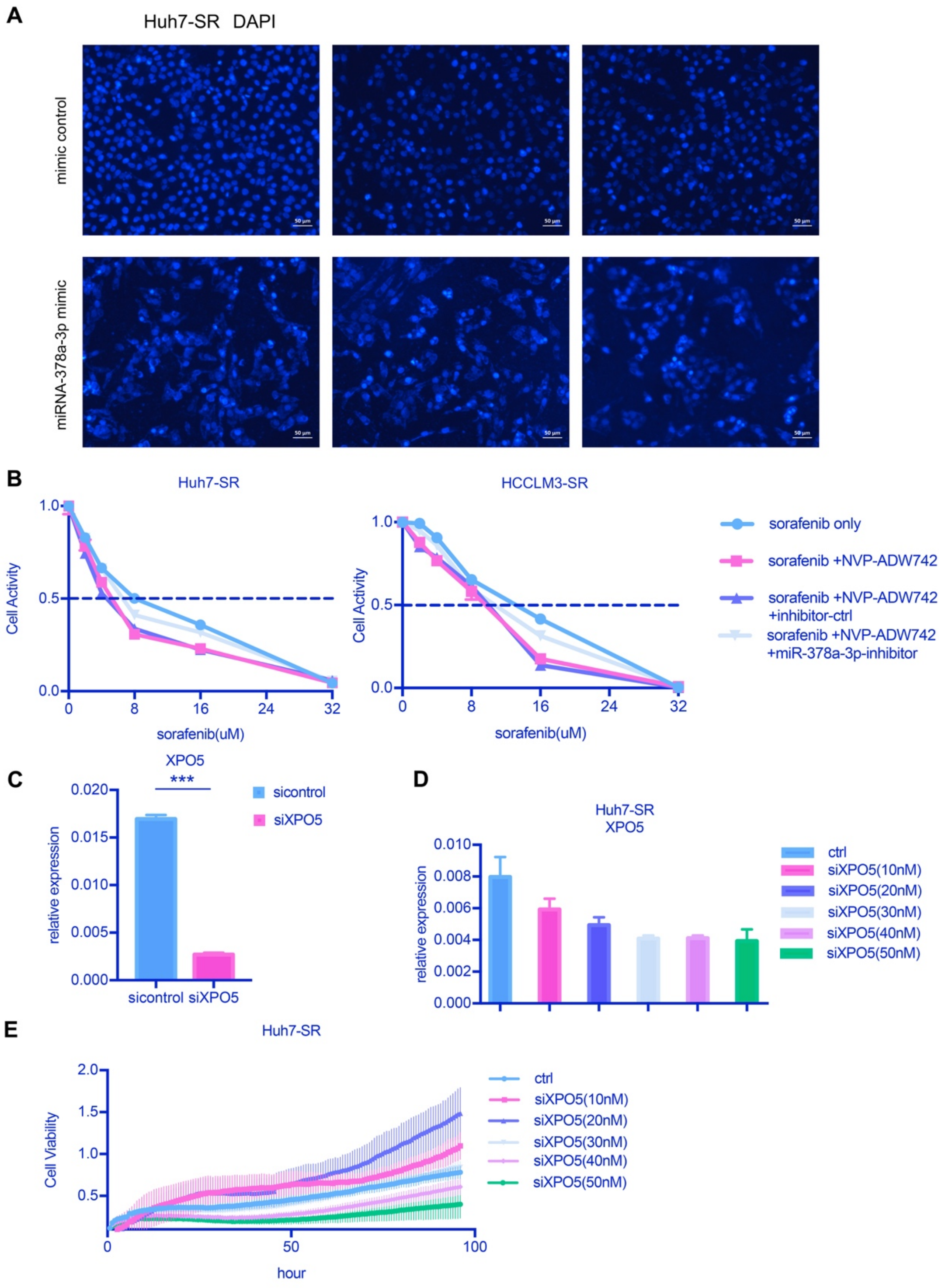

Figure S1. (A) Fluorescence microscopy image post-miR-378a-3p transfection and incubation with sorafenib for 48 h; (B) IGF1R inhibitor (NVP-ASW742) significantly blocked the miR-378a-3p inhibitor-mediated sorafenib resistance of HCC cells; (C) si-XPO5 did knockdown the XPO5. (D) The expression of XPO5 under transfected different concentration of siRNA; (E) The viability of Huh7-SR cells under transfected different concentration of siRNA.

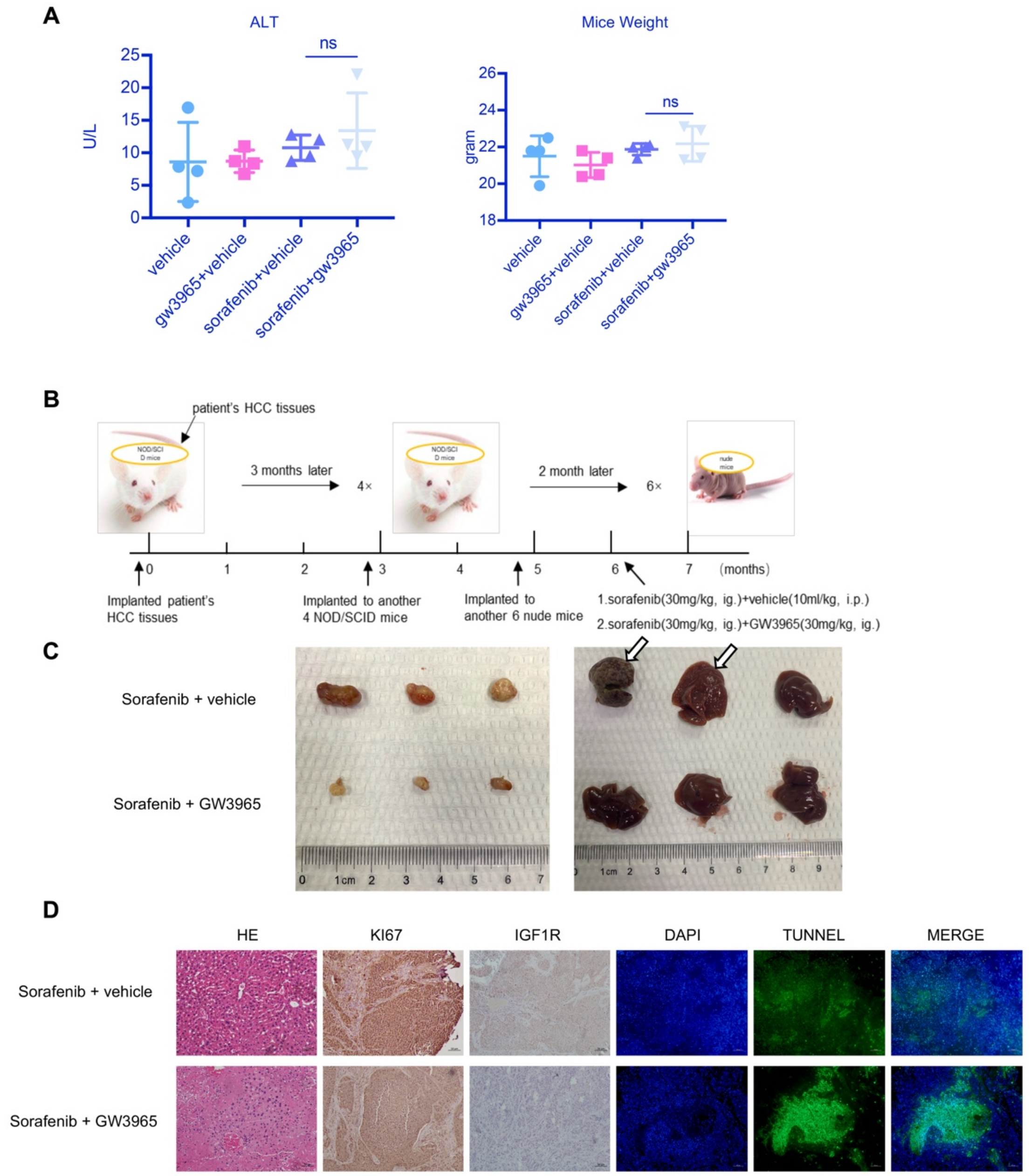

Figure S2. (A) the ALT level of four groups; the weight of four groups; (B) Schematic representation of the PDX model timeline; (C) gross view of tumors from two groups; Liver view of tumors from two groups; (D) Immunohistochemistry showed combination therapy could suppress HCC metastasis, proliferation, IGF1R level and TUNEL assay in PDX model.

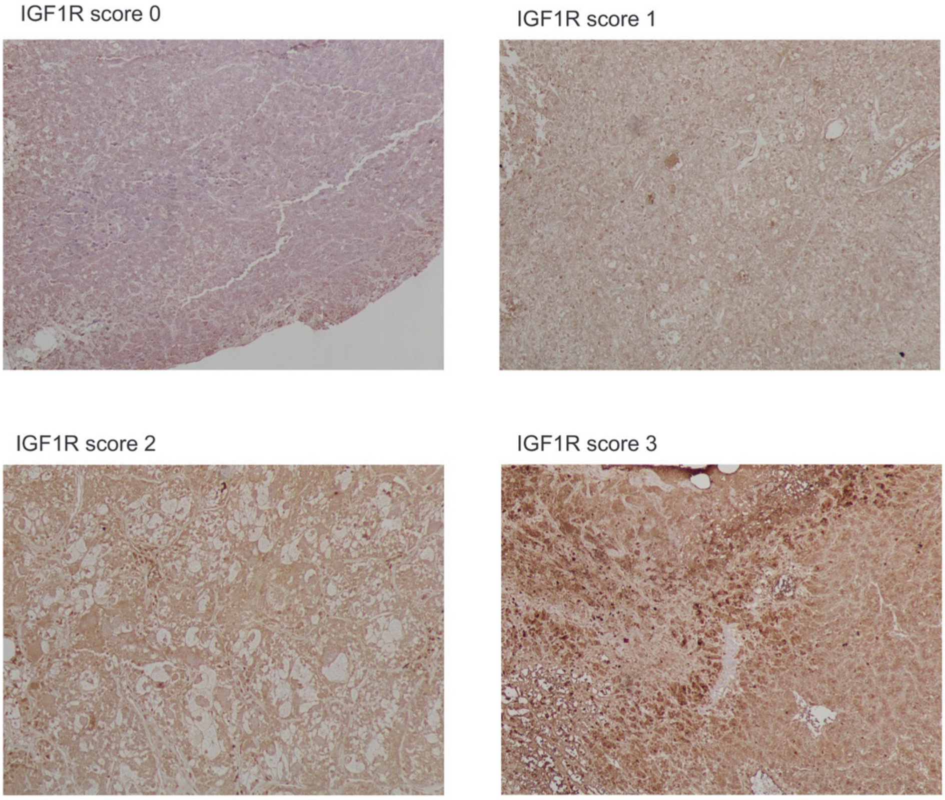

	High IGF1R	Low IGF1R	
Tumoral tissue	37	63	100

	High miR-378a-3p	Low miR-378a-3p	
Tumoral tissue	41	59	100

Figure S3. We used IHC experiment to measure the expression level of IGF1R in the 100 tumoral samples. The expression level was rated as 0 for negative, 1 for mild positive, 2 for positive, 3 for severe positive. Score 0 and 1 were considered as low expression, while score 2 and 3 were included in high expression.
